# The Potential Impacts of a Digital Preoperative Assessment Service on Appointments, Travel-Related Carbon Dioxide Emissions, and User Experience: Case Study

**DOI:** 10.2196/28612

**Published:** 2022-02-16

**Authors:** Madison Milne-Ives, John Leyden, Inocencio Maramba, Arunangsu Chatterjee, Edward Meinert

**Affiliations:** 1 Centre for Health Technology University of Plymouth Plymouth United Kingdom; 2 Ultramed Ltd Truro United Kingdom; 3 Department of Primary Care and Public Health School of Public Health Imperial College London London United Kingdom; 4 Harvard T H Chan School of Public Health Harvard University Boston, MA United States

**Keywords:** preoperative care, preoperative period, telemedicine, telehealth, appointments, cost-effective, economic

## Abstract

**Background:**

The National Health Service (NHS) cannot keep up with the demand for operations and procedures. Preoperative assessments can be conducted on the internet to improve efficiency and reduce wait times for operations. MyPreOp is a cloud-based platform where patients can complete preoperative questionnaires. These are reviewed by a nurse who determines whether they need a subsequent face-to-face appointment.

**Objective:**

The primary objective of this study is to describe the potential impact of MyPreOp (Ultramed Ltd) on the number of face-to-face appointments. The secondary objectives are to examine the time spent on preoperative assessments completed using MyPreOp in NHS Trusts and user ratings of usability and acceptability.

**Methods:**

The study design was a case study service evaluation. Data were collected using the MyPreOp system from 2 NHS Trusts (Guy’s and St Thomas’ and Royal United Hospitals Bath) and the private BMI Bath Clinic during the 4-month period from September to December 2020. Participants were adults of any age and health status at the participating hospitals who used MyPreOp to complete a preoperative assessment before a scheduled surgery. The primary outcome was the number of face-to-face appointments avoided by patients who used MyPreOp. The investigated secondary outcomes included the length of time spent by nurses completing preoperative assessments, associated travel-related carbon dioxide emissions compared with standard care, and quantitative user feedback. User feedback was assessed at all 3 sites; however, the other outcomes could only be examined in the Royal United Hospitals Bath sample because of data limitations.

**Results:**

Data from 2500 participants were included. Half of the assessed patients did not need a further face-to-face appointment and required a median of only 5.3 minutes of nurses’ time to review. The reduction in appointments was associated with a small saving of carbon dioxide equivalent emissions (9.05 tons). Patient feedback was generally positive: 79.8% (317/397) of respondents rated MyPreOp as easy or very easy to use, and 85.2% (340/399) thought the overall experience was good or very good.

**Conclusions:**

This evaluation demonstrates the potential benefits of MyPreOp. However, further research using rigorous scientific methodology and a larger sample of NHS Trusts and users is needed to provide strong evidence of MyPreOp’s efficacy, usability, and cost-effectiveness.

## Introduction

### Background and Rationale

The UK National Health Service (NHS) is unable to keep up with the demand for operations and procedures; it has failed to meet its 18-week waiting time goal since 2016 [1-3]. Preoperative assessments are essential to mitigate patient risk during surgery and support their recovery [4-7]. However, across the NHS, these assessments are predominantly administered using nonstandard, paper-based questionnaires [8-10]. With >10 million operations and procedures occurring each year [11,12], conducting these assessments to a high standard is time-intensive. The Royal College of Anaesthetists (RCoA) recommends 30- to 45-minute appointments; however, preoperative assessments can take up to 2 hours [6,8,13-15]. Health care staff often need to manually transfer the data collected into hospital information technology systems, which introduces another opportunity for error and hinders rapid screening of patients [16]. The Digital by Default report determined that preoperative assessments could be conducted remotely in 40% of cases, eliminating 1.2 million appointments and saving up to £48 (US $76) million [17]. Therefore, reducing the need for nurses and health care assistants to collect patient health records would be significantly valuable in terms of saving both time and cost.

### Solution Overview

MyPreOp (Ultramed Ltd) is a cloud-based platform that empowers patients to complete preoperative assessments on the web, thereby improving data quality, streamlining admission procedures, and ultimately saving time and costs [18]. Patients can complete the questionnaire in their own time and choose to share their data with their health care provider (retaining ownership). MyPreOp uses decision-support algorithms to determine what questions to ask depending on patients’ previous responses (reducing the number of questions they have to complete), to analyze the data to determine the American Society of Anesthesiologists (ASA) grade of patients [19], and to recommend the National Institute for Health and Care Excellence–guided preoperative tests [20]. The data and analysis are currently reviewed by a registered nurse in MyPreOp’s clinician portal, and the patient is moved along the appropriate care pathway.

MyPreOp is hosted on Google Cloud [21] and is compliant with Fast Healthcare Interoperability Resources (FHIR) Health Level 7 standards of interoperability [22,23], so the preoperative assessment report can be easily incorporated into patients’ electronic health records. MyPreOp automatically codes data using the Systematized Nomenclature of Medicine–Clinical Terms (SNOMED CT) [24,25] and generates International Classification of Diseases–10 (ICT-10) codes for comorbidities [26,27], providing a standardized clinical summary.

### Potential Benefits of Solution

MyPreOp has the potential to provide several key benefits for patients, clinicians, and health systems. It can provide patients with control over their personal health records and could improve the patient experience by increasing convenience, minimizing hospital visits, and decreasing the need to discuss sensitive topics. MyPreOp also includes built-in links to provide patients with easy access to accurate information about their procedure. Clinical benefits could include reducing the time clinicians spend conducting assessments and analyzing data, allowing them to spend more time on high-value care activities.

The use of digital preoperative assessments could also have significant economic benefits for health systems. According to RCoA requirements, conducting 12,000 preoperative assessments currently requires 7.2 whole time equivalent (WTE) nurses and 3.6 WTE health care assistants [6]. In comparison, a preoperative assessment service using MyPreOp requires about 3.7 WTE nurses and 1.1 WTE health care assistants. After including the costs for MyPreOp [28], this represents a potential 38% reduction in service costs. By enabling home completion of preoperative assessments, MyPreOp is also likely to reduce travel costs for the patient (and carers) and environmental costs from that travel.

### Aims and Objectives

This study aims to evaluate the potential of MyPreOp (Ultramed Ltd) to provide clinical and economic benefits when replacing the current standard of care. Specifically, the aim is to investigate the impact of the MyPreOp system on the time and environmental costs associated with preoperative assessments in 1 clinical site where it has been adopted and to examine ratings of its usability and acceptability in 3 clinical sites. The objectives of this case study are as follows:

1. Measure the time saved through the use of MyPreOp by assessing the number of face-to-face appointments avoided and the time spent by nurses completing the MyPreOp process at Royal United Hospitals Bath (RUHB) NHS Trust
2. Estimate the reduction in travel and associated carbon dioxide (CO_2_) emissions because of the reduction in face-to-face appointments at RUHB NHS Trust
3. Examine quantitative feedback about MyPreOp from users in 3 clinical sites (RUHB NHS Trust, Guy’s and St Thomas’ [GSTT] NHS Trust, and BMI Bath Clinic)
4. Compare patient responses to questions about the usability of MyPreOp with a previous service evaluation

## Methods

### Study Design

This investigation used a case study design (Table 1) to perform a formative service evaluation of data collected during the use of MyPreOp at 2 NHS Trusts and a private hospital. A case study framework [29] was used to structure the process of the evaluation. A formative service evaluation [30] was conducted to assess how well MyPreOp achieves its main aim of streamlining the preoperative assessment process in its early implementation [31]. This will provide preliminary evidence to inform future clinical investigations and cost analyses of the MyPreOp system. As the data used were collected and anonymized by a second party with informed consent, formal ethical approval for this evaluation was unnecessary.

**Table 1:** Case study framework [29,32,33].

Number	Stage	Outcome
1	Plan	Description of problem, case, and research questions
2	Design	Construction of case study design and linkage of research questions and available data
3	Prepare	Selection of NHS^a^ Trusts with appropriate data and sufficient sample sizes
4	Collect	Collection of MyPreOp use and patient feedback data from the MyPreOp analytics dashboards and the MyPreOp system
5	Analyze	Descriptive analysis and validation of data
6	Create	Drafting of the case study (this paper)
7	Share	Submission of the case study for publication in a peer-reviewed journal (this paper)

^a^NHS: National Health Service.

### Context and Participants

This study evaluated version 2 of MyPreOp. Versions 1 and 2 are similar from a patient perspective; however, version 2 is FHIR–based and cloud-based and includes a clinician portal. A total of 2 NHS Trusts using version 2 were included in this study: RUHB [34] and GSTT [35]. Data from the private BMI Bath Clinic were also included in the analysis of user feedback [36]. These hospitals were selected as they had used the MyPreOp system with the largest number of patients and had the most data available for analysis per site, and as they had the specific customizations and collaborations needed to collect the relevant data. These included the system being set up to ask relevant user feedback questions, statuses within the system that facilitated user feedback, statistics about face-to-face appointments, and an understanding of how the clinical sites’ processes aligned with the statuses being entered into the system (so the face-to-face appointment data could be verified). The other hospitals that used MyPreOp version 2 were excluded because of low numbers of submissions (n<300) or a high degree of customization, meaning relevant data could not be collected. Most of the analysis was conducted on data from RUHB, as they have been using MyPreOp version 2 for a longer period than the other sites and, therefore, have the largest body of service data.

All available patient submissions on MyPreOp during the study period were included in the analysis, regardless of age, health status, or type of surgery so the analysis would reflect typical patient use. However, the number of submissions included for each specific analysis varied depending on certain factors, such as whether the nurse had marked the submission as complete or whether the patient had answered a specific question.

### Data Collection

Anonymized operational data were collected from and processed by the MyPreOp system at each of the clinical sites for a 4-month period from September 1, 2020, to December 31, 2020. One of the authors (JL) created data sets from the raw JSON data using BigQuery SQL and manually examined a small subset of data to check that it was being processed correctly.

Raw data were automatically collected and compiled using the MyPreOp system. Clinicians use their MyPreOp portal to set patients’ status as they move through the process (eg, requiring a face-to-face appointment with a nurse or anesthetist). The number of avoided face-to-face appointments was assumed to be the number of patients who progressed through the entire process without having their status set to requiring a face-to-face appointment. The system also tracks the length of time from the start of nurses’ processing of a patient on MyPreOp to the assessment being uploaded into the patient’s record.

The amount of carbon emissions saved by using MyPreOp was calculated from patient-reported data about their distance from the hospital (in miles) and the mode of transit they usually use to travel to the hospital (car, motorcycle, bus, train, bicycle, or walking), although these data were only available for RUHB, as the other sites chose to ask their patients different questions. Patients who did not need face-to-face appointments were assumed to have avoided one return trip to the hospital. A carbon footprint calculating website [37] was used to calculate the approximate CO_2_ equivalent (CO_2_e) of the travel avoided by using MyPreOp.

User feedback data were collected from patient feedback questions presented at the end of the MyPreOp questionnaire and stored in the MyPreOp system.

### Data Analysis

A descriptive analysis was conducted by one of the authors (JL) to summarize the data collected. The same author created visualizations of the data in DataStudio (Google). No statistical analyses were conducted because of the limitations of the study design and the collected data. The service evaluation at RUHB identified the percentage of patients who were not listed as requiring face-to-face follow-up appointments, the mean and median of nurse time spent on assessments, and an estimate of CO_2_ emissions avoided by reducing the number of patients seen for face-to-face appointments. Usability data collected from the 3 clinical sites examined in this study were summarized and compared with a previous service evaluation of MyPreOp in different NHS Trusts [38].

## Results

### Overview

During the 4-month period of data collection (September 1, 2020, to December 31, 2020), there were 2500 MyPreOp submissions from patients at the three clinical sites: 71.08% (n=1777) were from patients at RUHB, 16.24% (n=406) were from GSTT, and 12.68% (n=317) were from BMI Bath. The total number of patients assessed for each outcome measure is reported for the individual analyses, as it does not always equal the total number of submissions. This is because patients were not required to answer all questions, and not all submissions had progressed through the whole system to completion at the time of data collection.

### Face-to-face Appointments Avoided

**Figure 1:**
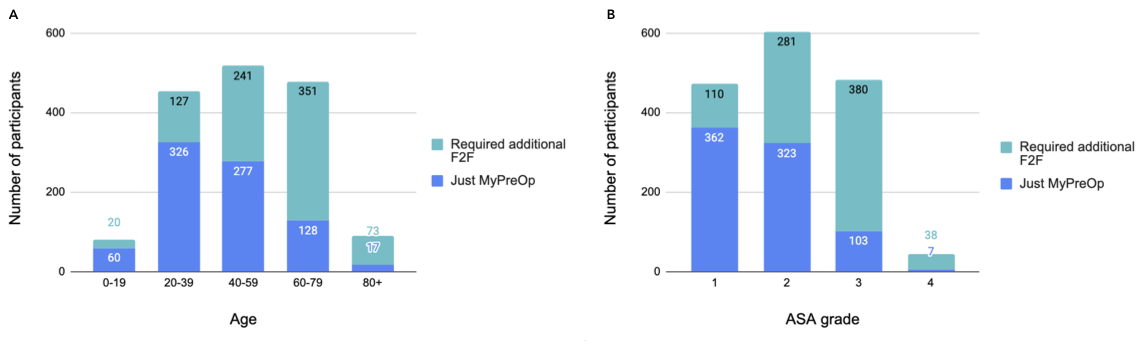
Proportions of (A) patients needing face-to-face appointments by age and (B) American Society of Anesthesiologists grade (data from the Royal United Hospitals Bath). ASA: American Society of Anesthesiologists; F2F: face-to-face.

Of the patients who used the MyPreOp assessment at RUHB during the 4-month period, half (813/1630, 49.88%) did not require any further face-to-face follow-up. The total sample for this analysis included patients who completed the assessment and those who had been flagged on the system as requiring a face-to-face assessment but had not yet had the appointment. It excluded patients whose preoperative assessments had not yet been processed. The number of patients requiring face-to-face appointments varied by age and ASA grade (Figure 1). The totals differed slightly, as a small minority of patients who did not have their age or ASA grade correctly entered into the system were excluded from the analysis. There was a greater number of patients aged <60 years who did not require a face-to-face appointment (663/1051, 63.08%) than those who did (388/1051, 36.92%), although this was more pronounced at younger ages. A similar trend was observed for ASA grades, with more patients with lower ASA grades (1 and 2) avoiding face-to-face appointments than those with higher grades. Data on face-to-face appointments avoided for GSTT and BMI Bath could not be included in this analysis, as the process of nurses flagging the patients who required a face-to-face assessment on the system could not be fully validated throughout the entire trial period, unlike with RUHB.

### Nursing Time Spent Completing MyPreOp

The distribution of time that nurses at RUHB spent completing MyPreOp assessments for patients who did not require a face-to-face appointment was heavily skewed to the short side. The median amount of time nurses spent completing assessments was 5.3 (IQR 3.2-12.9) minutes and the mode was 2 minutes; significantly shorter than the mean time of 49.9 minutes (SD 454.7 minutes; n=860). The data were skewed heavily to the right by the inclusion of a small percentage of assessments that had a long time between start and completion (94/860, 10.9% of assessments took nurses longer than an hour to complete). If those assessments were excluded, the mean time to complete the assessment was 6.8 minutes (SD 7.4 minutes; n=766). However, as time spent on the assessment was measured by the difference between when it began and when it was marked as complete, the cause of these delays could not be accounted for in this analysis.

### CO_2_ Reduction

The vast majority of RUHB patients (1583/1757, 90.1%) used a car as their usual mode of transit to the hospital. Half of the respondents (771/1541, 50.03%) lived between 5 and 15 miles away from the hospital, about a third (517/1541, 33.55%) lived >15 miles away from the hospital, and the remaining 16.42% (253/1541) lived within 5 miles of the hospital. Information about patients’ usual mode of transit was combined with their distance from the hospital (Table 2) and the data on the number of avoided appointments to calculate potential carbon savings. Over the 4-month period, the reduction in face-to-face appointments at RUHB is estimated to have resulted in a total carbon savings of 9.05 tons of CO_2_e.

**Table 2:** Distance that patients need to travel to get to Royal United Hospitals Bath hospitals stratified by mode of transit (N=1541).

Mode of transport	Distance from hospital (miles), n (%)		
	0-5	5-15	≥15
Car (n=1402)	177 (13)	732 (52)	493 (35)
Bus (n=56)	17 (30)	27 (48)	12 (21)
Train (n=16)	1 (6)	4 (25)	11 (69)
Motorcycle (n=2)	1 (50)	1 (50)	0 (0)
Bicycle (n=8)	5 (63)	3 (38)	0 (0)
Walk (n=57)	52 (91)	4 (7)	1 (2)
Total (n=1541)	253 (16)	771 (50)	517 (34)

### User Feedback

User feedback was examined using data from both NHS Trusts (RUHB and GSTT) and the private BMI Bath Clinic. Across the 3 sites, 87.94% (2195/2496) of patients reported completing MyPreOp on their own. Of the patients who reported having assistance completing MyPreOp, only 3.8% (10/266) were helped by a member of staff; the remaining patients were assisted by relatives, friends or neighbors, or parents or guardians. To facilitate the evaluation and improvement of MyPreOp, patients were also asked if they consented to have their anonymized data used for research. Of those who responded (from GSTT, RUHB, and BMI Bath), 81.89% (1741/2126) said that they were happy for their anonymized data to be used.

As the clinical sites did not all use the same user feedback questions, the remaining analyses were conducted separately for each site data set. BMI Bath assessed the length of time the patients required to complete MyPreOp. Nearly half of the patients completed MyPreOp within ≤30 minutes (131/301, 43.5%), and less than a quarter of patients needed >45 minutes (65/301, 21.6%).

At GSTT, a total of 403 patients completed MyPreOp assessments over the 4-month period. Most of these patients responded to the patient feedback questions provided at the end of the MyPreOp questionnaire; however, they were not mandatory, so the number of respondents varied per question. MyPreOp was generally rated highly on user feedback: 79.8% (317/397) rated MyPreOp as easy or very easy to use, with only 6.3% (25/297) finding it difficult or very difficult to use, and 85.2% (340/399) thought the overall experience was good or very good with only 3.3% (13/399) rating it as poor or very poor. At RUHB, patients were asked if they had any concerns about MyPreOp; 88.1% (1548/1757) reported having none.

Users at GSTT were also asked to provide feedback on the additional supporting information provided by the system. Furthermore, 82.9% (320/386) of patients thought that the information provided by MyPreOp about what to expect next in their preoperative pathway was somewhat or very easy to understand, and 80.6% (312/387) of patients rated the additional health information provided as quite or very useful.

## Discussion

### Principal Findings

The data from the RUHB NHS Trust demonstrated that half of the patients who used the MyPreOp service for their preoperative assessment did not require a face-to-face appointment. This is higher than the Digital by Default’s 2012 estimate that 40% of secondary care preoperative appointments could be avoided by using remote screening [17] but will need to be confirmed in larger, more diverse samples. The reduction in appointments was most prominent in users who were younger and healthier (as indicated by a low ASA score). Therefore, the impact of the service could be limited, as younger and healthier patients might be more likely to have more straightforward and rapid preoperative assessments.

A reduction in preoperative assessment appointments has the potential to save nurses’ time. The RCoA recommends that preoperative assessments be scheduled to last 30 minutes (for day patients) to 45 minutes (for inpatients) [6]. According to the time logs from the MyPreOp data, nurses at RUHB spent a median of approximately 5 minutes on patients who did not need a face-to-face appointment. During the period of data collection, 49.88% (813/1630) of the patients at RUHB avoided an appointment. If the time spent on an average patient is 33 minutes (the RCoA assumes a ratio of 80% day patients and 20% inpatients [6]), and the median time spent on patients who avoided an appointment is 5 minutes, an estimate of the average time saved for each of those 813 patients was 28 minutes. In this sample, this would represent approximately 379 hours saved. Although this estimation is based on a relatively small sample, it illustrates MyPreOp’s potential to reduce the time nurses spend on preoperative assessments. However, over half of the users reported needing at least 30 minutes to complete their assessment, so potential time savings for patients appear to be more limited. These findings should be examined in a clinical trial to establish further evidence of the impact of MyPreOp on time spent on preoperative assessments.

A reduction in face-to-face appointments also has the potential to reduce travel, which could save time for patients and contribute to reducing carbon emissions. The amount of carbon savings identified in this study (9.05 tons) is small compared with the United Kingdom’s net CO_2_ emission (351.5 million tons in 2019) [39]. However, transport is the biggest contributor to CO_2_ emissions in the United Kingdom (34% in 2019) [39,40], with road transport (particularly passenger cars) accounting for the largest proportion of emissions in that sector [41,42]. Therefore, reducing car use is one of several key strategies for reducing transport-related carbon emissions [43,44]. Although any preoperative assessment–related travel reductions associated with remote preoperative assessments will not be a large proportion of road transport, it is aligned with the NHS’s net zero carbon goal [45].

Overall, most patients at GSTT rated MyPreOp fairly positively on the user feedback questions. These results are similar to a previous service evaluation of MyPreOp version 1 (unpublished data), which found high ratings of overall experience (974/1193, 81.64% rated it as good or excellent) and ease of use (1119/1193, 93.8% thought it was very easy or easy enough to use) [38]. The data assessed from GSTT in this service evaluation found a slightly lower rating for ease of use (317/397, 79.9% rated MyPreOp as easy or very easy to use). The wording of the usability questions varied slightly between the 2 evaluations (*very easy* or *easy* in this assessment compared with *very easy* or *easy enough* in the previous one), which could have affected ratings. However, the variation seems to come from fewer people rating MyPreOp as *very easy* in this assessment (173/397, 43.6%) compared with the previous one (697/1193, 58.43%); ratings for *easy* (144/397, 36.3%) and *easy enough* (422/1193, 35.37%) were similar. It is possible that sample demographics influenced the ratings, and research in larger and more diverse samples will be necessary to explore potential demographic differences in acceptability and usability further to evaluate any potential impact of MyPreOp on health inequalities.

### Limitations of the Study

A major limitation on the interpretability of the study is that it was a service evaluation without a rigorous, pre-established methodology or statistical analysis. To mitigate this, the Standards for Reporting Qualitative Research checklist was used in the preparation of this paper (Multimedia Appendix 1 [46]). However, it cannot provide strong evidence of any positive or negative impacts of MyPreOp on the outcomes examined and only demonstrates the feasibility of the solution and its potential impacts. A controlled clinical trial is necessary to provide evidence of the efficacy of MyPreOp in reducing the time, economic, and environmental costs of preoperative assessments.

The data were provided to the academic team in a processed form because of difficulties and concerns about accessing the Ultramed system. One author (JL) used SQL queries to extract JSON data into tables. This introduces a potential for bias and conflict of interest, as the quality of the data depends on the accuracy of those queries, which were not validated by a second author.

The measure of avoided face-to-face appointments is limited, as it uses the patient statuses set by nurses in MyPreOp as an indicator of whether the patient had a face-to-face appointment. There was no external validation of the accuracy of these statuses and whether the patient actually avoided a face-to-face appointment.

Another limitation is that the data were only available for individual NHS Trusts for most of the outcomes measured. A compilation of data from each of the Trusts would have provided larger samples from more diverse populations. For example, many of the patient feedback questions included at the end of the MyPreOp questionnaires varied depending on the Trust and could not be collated. This raises another limitation: the user feedback questions displayed at the end of MyPreOp were selected by the individual Trusts and based on what they perceived to be most useful to them, not a usability theory or framework. The lack of a theoretical framework and validated measure, as well as the difference in wording between Trusts, introduce potential bias in the evaluation of usability and acceptability.

### Future Directions

Further research is needed to examine the cost and time benefits of MyPreOp on a larger scale. This should be conducted as a proper academic study and include a full health economic assessment (including environmental costs) instead of a service evaluation, as a pre-established methodology will increase the credibility of the results. A comparison of the time and costs of using MyPreOp compared with current standards of care would also provide a more compelling argument for the use of digital preoperative assessment services in general and MyPreOp in particular [47,48].

More research into patient usability would also be beneficial [49]. Future studies should include a theory-based qualitative examination of patient feedback regarding acceptability and usability. This will likely be particularly important for older users, as there is an increasing number of older adults undergoing surgery [50], and there tends to be a greater digital exclusion of older people [51,52]. Evaluating the usability of digital health solutions in older adults—and other groups who might struggle to access digital services—is important to ensure that MyPreOp and other digital solutions do not worsen existing health inequalities.

### Conclusions

The aim of this evaluation was to describe the data being collected by MyPreOp and to provide an assessment of the potential benefits of its implementation. From the data included in this study, a reduction in the number of face-to-face appointments was observed; however, this appeared to vary depending on age and ASA grade. A potential reduction in the time spent on preoperative assessments that did not require a face-to-face appointment was observed for nurses but not for patients. The reduction in face-to-face appointments was demonstrated to have a potential impact on travel-related CO_2_e emissions. The study also found generally positive ratings for MyPreOp. However, the quantity and quality of the evidence, as well as the methodology of this service evaluation, are not sufficient to provide strong support for the efficacy and usability of MyPreOp. Further studies should be conducted using rigorous scientific methods and including more clinical sites to evaluate a greater range of outcomes, including cost-effectiveness, compared with the current standard of care and qualitative user feedback.

## Appendix

Multimedia Appendix 1 Standards for Reporting Qualitative Research checklist [46].

## Abbreviations

ASA: American Society of Anesthesiologists
CO_2_e: CO_2_ equivalent
GSTT: Guy’s and St Thomas’
NHS: National Health Service
RCoA: Royal College of Anaesthetists
RUHB: Royal United Hospitals Bath
WTE: whole time equivalent

## References

[1] Baker C (2021). NHS Key Statistics: England, October 2021. House of Commons Library.

[2] (2019). NHS performance and waiting times. The Health Foundation.

[3] (2019). NHS pressures in England: waiting times, demand, and capacity. House of Commons Library.

[4] Swart M, Houghton K (2010). Pre-operative preparation: essential elements for delivering enhanced recovery pathways. Curr Anaesthesia Crit Care.

[5] Zambouri A (2007). Preoperative evaluation and preparation for anesthesia and surgery. Hippokratia.

[6] (2019). Chapter 2: guidelines for the provision of anaesthesia services for preoperative assessment and preparation 2019. Royal College of Anaesthetists.

[7] García-Miguel F, Serrano-Aguilar P, López-Bastida J (2003). Preoperative assessment. The Lancet.

[8] Online preoperative assessment. NHS Networks.

[9] Hawes RH, Andrzejowski JC, Goodhart IM, Berthoud MC, Wiles MD (2016). An evaluation of factors influencing the assessment time in a nurse practitioner-led anaesthetic pre-operative assessment clinic. Anaesthesia.

[10] Before surgery -Having an operation (surgery). NHS.

[11] (2017). Key statistics on the NHS. NHS Confederation.

[12] Abbott T, Fowler A, Dobbs T, Harrison E, Gillies M, Pearse R (2017). Frequency of surgical treatment and related hospital procedures in the UK: a national ecological study using hospital episode statistics. Br J Anaesth.

[13] Pre-op assessment. Northern Care Alliance NHS Foundation Trust.

[14] Pre-assessment. The Royal Marsden NHS Foundation Trust.

[15] Information for patients attending the Pre Operative Assessment Clinic. Oxford Radcliffe Hospitals NHS Trust.

[16] Ultramed. Mylor Ventures.

[17] Digital by default: the delivery choice for England’s population. Innovation and Health.

[18] My PreOp. Ultramed.

[19] (2020). ASA physical status classification system. American Society of Anesthesiologists.

[20] (2016). Routine preoperative tests for elective surgery. National Institute for Health and Care Excellence.

[21] Cloud computing services. Google Cloud.

[22] Welcome to FHIR. HL7FHIR.

[23] Fast healthcare interoperability resources. NHS Digital.

[24] SNOMED home. SNOMED International.

[25] Overview of SNOMED CT. National Library of Medicine.

[26] International Statistical Classification of Diseases and Related Health Problems 10th Revision. ICD-10 Version:2010.

[27] International Statistical Classification of Diseases and Related Health Problems (ICD). World Health Organization.

[28] Ultramed's Ultraprep - pre procedure assessments. GOV.UK Digital Marketplace.

[29] Yin R (2017). Case Study Research and Applications Design and Methods.

[30] Bowling A (2014). Research Methods in Health Investigating Health and Health Services.

[31] Moule P, Armoogum J, Dodd E, Donskoy A, Douglass E, Taylor J, Turton P (2016). Practical guidance on undertaking a service evaluation. Nurs Stand.

[32] van Velthoven MH, Lam C, de Cock C, Stenfors T, Chaudhury H, Meinert E (2020). Development of an innovative real-world evidence registry for the herpes simplex virus: case study. JMIR Dermatol.

[33] Meinert E, Milne-Ives M, Surodina S, Lam C (2020). Agile requirements engineering and software planning for a digital health platform to engage the effects of isolation caused by social distancing: case study. JMIR Public Health Surveill.

[34] Royal United Hospitals Bath. NHS Foundation Trust.

[35] Guy's and St Thomas' NHS Foundation Trust homepage. Guy's and St Thomas' NHS Foundation Trust.

[36] BMI Bath Clinic. BMI Healthcare.

[37] Carbon footprint calculator. carbonfootprint.org.

[38] Maramba I, Chatterjee A (2021). Continuous user experience monitoring of a patient-completed preoperative assessment system in the United Kingdom: cross-sectional study. JMIR.

[39] (2020). 2019 UK greenhouse gas emissions, provisional figures. Department for Business, Energy, and Industrial Strategy.

[40] Stark C, Thompson M, Andrew T, Bellamy O, Cole C, Darke J, Davies E, Gault A, Goater A, Hay R, Hemsley M, Hill J, Joffe D, Kmietowicz E, Livermore S, Millar R, Nemo C, Scudo A, Thillainathan I, Vause E (2019). Reducing UK emissions: 2019 Progress Report to Parliament. Committee on Climate Change.

[41] (2020). 2018 UK greenhouse gas emissions, final figures. Department for Business, Energy, and Industrial Strategy.

[42] (2019). Road transport and air emissions. Office for National Statistics.

[43] Routes to lower greenhouse gas emissions transportation future. United States Environmental Protection Agency.

[44] Reducing your transportation footprint. Center for Climate and Energy Solutions.

[45] (2020). Delivering a ‘net zero’ national health service. National Health Service.

[46] O'Brien BC, Harris IB, Beckman TJ, Reed DA, Cook DA (2014). Standards for reporting qualitative research: a synthesis of recommendations. Acad Med.

[47] (2006). Cost-effectiveness analysis. Priorities in Health.

[48] An overview of the rationale, activities and goals of WHO-CHOICE. World Health Organization.

[49] Carayon P, Hoonakker P (2019). Human factors and usability for health information technology: old and new challenges. Yearb Med Inform.

[50] Fowler AJ, Abbott TE, Prowle J, Pearse RM (2019). Age of patients undergoing surgery. Br J Surg.

[51] Davidson S (2018). Digital inclusion evidence review 2018. Age UK.

[52] Helsper EJ, Reisdorf BC (2016). The emergence of a “digital underclass” in Great Britain and Sweden: changing reasons for digital exclusion. New Media Soc.

